# Endothelial Cell Distribution After Flow Exposure With Two Stent Struts Placed in Different Angles

**DOI:** 10.3389/fphys.2021.733547

**Published:** 2022-01-13

**Authors:** Zi Wang, Narendra Kurnia Putra, Hitomi Anzai, Makoto Ohta

**Affiliations:** ^1^Institute of Fluid Science, Tohoku University, Sendai, Japan; ^2^Graduate School of Biomedical Engineering, Tohoku University, Sendai, Japan; ^3^Instrumentation and Control Research Group, Faculty of Industrial Technology, Institut Teknologi Bandung, Bandung, Indonesia

**Keywords:** endothelial cell, wall shear stress, flow chamber, stent strut, endothelialization, computational fluid dynamics

## Abstract

Stent implantation has been a primary treatment for stenosis and other intravascular diseases. However, the struts expansion procedure might cause endothelium lesion and the structure of the struts could disturb the blood flow environment near the wall of the blood vessel. These changes could damage the vascular innermost endothelial cell (EC) layer and pose risks of restenosis and post-deployment thrombosis. This research aims to investigate the effect of flow alterations on EC distribution in the presence of gap between two struts within the parallel flow chamber. To study how the gap presence impacts EC migration and the endothelialization effect on the surface of the struts, two struts were placed with specific orientations and positions on the EC layer in the flow chamber. After a 24-h exposure under wall shear stress (WSS), we observed the EC distribution conditons especially in the gap area. We also conducted computational fluid dynamics (CFD) simulations to calculate the WSS distribution. High EC-concentration areas on the bottom plate corresponded to the high WSS by the presence of gap between the two struts. To find the relation between the WSS and EC distributions on the fluorescence images, WSS condition by CFD simulation could be helpful for the EC distribution. The endothelialization rate, represented by EC density, on the downstream sides of both struts was higher than that on the upstream sides. These observations were made in the flow recirculation at the gap area between two struts. On two side surfaces between the gaps, meaning the downstream at the first and the upstream at the second struts, EC density differences on the downstream surfaces of the first strut were higher than on the upstream surfaces of the second strut. Finally, EC density varied along the struts when the struts were placed at tilted angles. These results indicate that, by the presence of gap between the struts, ECs distribution could be predicted in both perpendicular and tiled positions. And tiled placement affect ECs distribution on the strut side surfaces.

## Introduction

Stent implantation is widely used to cure cardiovascular diseases such as stenosis or aneurysm (Borhani et al., 2018). However, during the stent deployment process, struts expansion could cause damage to the vascular endothelium. The endothelium lesion might induce first platelet aggregation to form a thrombus. Next, the overproliferated smooth muscle cells (SMCs) could generate neointima due to the inadequate endothelial cells (ECs) regulation. Multiple *in vivo* animal studies found that EC dysfunction could change the gene expression leading to abnormal platelet and SMC conditions (Kullo Iftikhar et al., 1997; Namba et al., 2003; Kipshidze et al., 2004; Songmingbao and Huanglan, 2014; Song and Lan, 2016). The formation of thrombus and neointima caused by EC dysfunction could promote the re-blocking of the blood vessel. The negative effect of re-blockage of the blood vessel, such as restenosis and thrombosis, has become a severe complication after the stenting treatment (Liu et al., 1989; Foley et al., 1994; Bavry et al., 2005; Mauri et al., 2005; Finn et al., 2007). Therefore, the complications by stent deployment could be considered as EC denudation around struts. To reduce the vessel re-blockage, accelerate the endothelialization, meaning covering the struts by ECs, is necessary as quick endothelialization on the strut could prevent platelet adhesion and inhibit SMC proliferation.

Endothelial cell forms the innermost layer in the blood vessel and is constantly exposed to the blood flow. To maintain vascular homeostasis, EC responds to the force generated by blood flow, especially the wall shear stress (WSS) (Jou et al., 2008; Meng et al., 2014; Cebral et al., 2017, 2019). Both *in vivo* and *in vitro* studies showed that WSS change could cause EC dysfunctions, such as those related to the vascular cell adhesion molecules secreted by ECs (Mannino et al., 2015; Kumar et al., 2018). On the morphological side, EC orientation and elongation could be observed due to the WSS change (Dewey et al., 1981; Levesque and Nerem, 1985; Palmer and Bizios, 1997).

The EC activities in response to the WSS drive the attention of *in vitro* study. The flow chamber could control the WSS on the lumen by its design. By using a parallel plate flow chamber, the inside flow condition could be set at a certain WSS value according to the inlet flow so that it is easier to subject the ECs to the WSS similar to the intravascular conditions (Usami et al., 1993; White Charles et al., 2001; Chung et al., 2003; McCann et al., 2005; Dolan et al., 2012; Balaguru et al., 2016; Wang et al., 2016; Erbeldinger et al., 2017). The parallel plate flow chamber is the most commonly used to study the flow stimuli on ECs. Various flow patterns could be induced in the chamber to study their effects on EC monolayer. In this study, the chamber was modified to make it capable to place the strut and allow the observation of EC on the strut surface. By the presence of one stent strut inside the flow chamber, the EC response distribution to the changed WSS due to the flow disturbance was observed (Anzai et al., 2020). Anzai et al. (2020) found that, after the flow exposure the appearance of ECs’ high concentration agrees with the appearance of high WSS using one strut. Including the paper (Anzai et al., 2020), several earlier studies on *in vitro* flow experiments have heavily utilized computational fluid dynamics (CFD) with a high degree of agreement toward the realistic flow conditions (Vogel et al., 2007; Kaur et al., 2012; Anisi et al., 2014).

On stent treatment, the intravascular flow conditions are usually predicted by CFD to deepen our understanding of how stenting affects intravascular hemodynamics and prove treatment efficacy (Frank et al., 2002). Predictions using CFD were also applied in the improvement of the stent design. Putra et al. (2017, 2019) showed that the gap between two struts influences the WSS environment as a stent design. The CFD could indicate different WSS distributions in the gap between struts. Then, the cell distributions on the strut surface in the gap should be observed.

Previous study used a parallel plate flow chamber with the presence of one strut observed EC distribution at both bottom plate of the dish and side surfaces of the strut. The objective of this study is to investigate the influence of gap between two struts on EC distribution at the dish surface and strut endothelialization process. We could observe that cell distribution follows the wall shear stress distribution by the CFD simulation. However, EC distribution on strut surfaces is different between the left and right sides, especially on side surfaces between the gap.

## Materials and Methods

In order to observe the EC distribution after the flow exposure with the presence of gap between two struts and the endothelialization effect on the surface of the strut, two struts were placed in a specific position and orientation on the EC monolayer inside the parallel flow chamber. CFD simulation was performed to analyze the flow condition inside the chamber.

### Experiment of Flow Exposure

The human carotid artery endothelial cells (HCtAEC, Cell Applications, Inc., United States) with passage numbers between 5 and 9 were used in this study. ECs were two-dimentionally cultured in 35-mm culture dishes precoated with gelatin solution (FUJIFILM Wako Pure Chemical Corporation, Japan) until reaching confluency. The proliferation medium used both in the cell culture and flow exposure experiments was the same as that described previously by Anzai et al. (2020).

**FIGURE 1:**
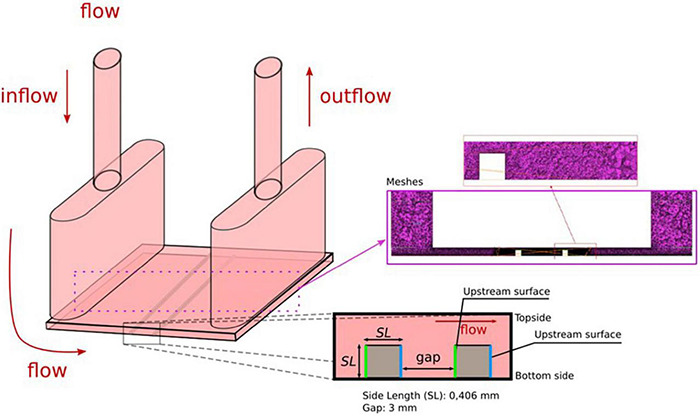
The geometry of computational fluid dynamics (CFD) simulation.

Figure 1 shows the geometry of the parallel flow chamber with the WSS exposure area of 22 mm × 18 mm referred to previous study (Anzai et al., 2020). NiTi stent strut (0.406 mm × 0.406 mm × 18 mm) (Furukawa Electric Co., Ltd., Japan) was placed and covered with silicone gaskets (Fusougomu, Japan) at the thickness of 5 mm to fix the strut positions during the experiment. One gasket sheet was prepared for 90°. The other one was 70° under the confirmation that both degrees could avoid the inlet flow effect. We also confirmed that WSS distribution of the 70° are relatively near to 60° compared to 90° using CFD. The outline of the stent struts and the flow exposure area (18 mm × 20 mm) on the silicone sheet were cut by using a digital cutter machine (FC4500-50, GRAPHTEC, Japan) to fit the 35 mm culture dishes.

The WSS in this study was 2 Pa, generated by a roller pump with a volume flow rate of 2.16 × 10^–6^ m^3^/s. To maintain the pH of the working fluid, gas (5% CO_2_ + 95% air) was connected to the flow loop. The temperature during the flow exposure was constantly kept at 37°C.

After 24 h of flow exposure, ECs were fixed in 4% paraformaldehyde for 15 min at room temperature, then rinsed in phosphate-buffered saline. To permeabilize the EC membrane, the cells were treated with 1-mL aliquots of 0.2% Triton X-100 (Roche Applied Science, Germany) for 5 min. The EC F-actin filaments and nuclei were stained with Alexa Fluor^®^ 546 Phalloidin (Gibco, United States) and 4’,6-diamidino-2-phenylindole (Gibco, United States), respectively. The fluorescent EC images were obtained using a microscope system (IX-83, Olympus, Japan).

Image processing was conducted using the ImageJ software (NIH). The EC density data were presented as the mean ± standard deviation (STD), where the mean represents the mean value of n measurements. The 90° and 70° experiments were performed with *n* = 6 and 3, respectively, to confirm reproducibility. The experimental data were analyzed using Student’s *t*-test to identify the levels of statistical significance (*p < 0.05) between the groups.

### Computational Fluid Dynamics Simulation

Figure 1 shows the shape of the parallel chamber used in the CFD simulation. The geometry of the fluid domain is based on the parallel flow chamber used in the EC *in vitro* experiment. Two identical struts, with the cross-sectional shape of 0.406 mm × 0.406 mm and 18 mm in length, were parallel-placed in the middle of the bottom surface. Previous study by Anzai et al. (2020) found the flow recirculation due to the strut disturbance. The distance of the flow re-attach point to the strut is within 2 mm. The second strut was placed as the gap distance of 3 mm to keep the re-attached point in the gap. The angle relative to the flow direction was set as 90° and 70°. The shape was constructed by SOLIDWORKS 2017 (Dassault Systèmes, Vélizy-Villacoublay, France), extracted to stereolithography (.STL) format, and imported later to the simulation program. The computational domain discretization was constructed by ANSYS ICEMCFD 17.2 (ANSYS Inc., Cannonsburgh, PA, United States) with a total of 22 million meshes consisting of tetrahedral and triangular elements. The mesh convergence test has been performed with the mesh element number ranging between 6.68 and 26 million elements to make assure the numerical solution accuracy. Finer mesh density was prescribed on the region of interest (ROI) around the strut placement area.

The CFD method has been performed by using ANSYS CFX (ANSYS Inc., Canonsburg, PA, United States) to estimate the flow phenomenon inside the flow chamber based on the Navier–Stokes solution. Since the EC culture media has similar fluid parameter with water, the flow media was considered as water with a density of 1,000 kg/m^3^ and a viscosity of 0.001 Pa⋅s. All solid domains were found as a rigid wall with non-slip boundary conditions. The flow conditions were defined as stationary flow with a volume rate inlet of 2.6 × 10^–6^ m^3^/s and a static pressure outlet at 0 Pa. The convergence criteria were set with 10^–5^ root to mean square residue.

The simulation process was carried out at the advanced fluid information facilities, Institute of Fluid Science, Tohoku University with eight cores and finished in 3 h and 30 min. The WSS distribution and flow pattern postprocessing analyses have been performed with ANSYS CFD-Post (ANSYS Inc., Canonsburg, PA, United States), and further data analysis has been completed with a data processing script executed by MATLAB (Mathworks Inc., Natick, MA, United States).

## Results

### The Distributions of EC and WSS in the Gap

**FIGURE 2:**
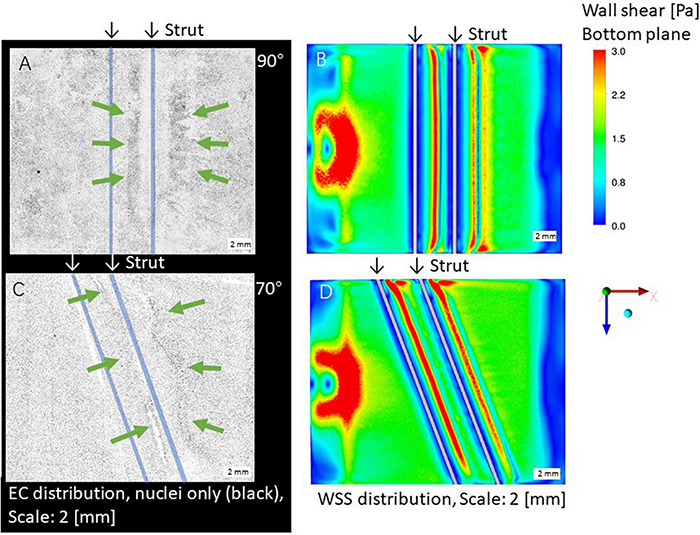
Distribution of endothelial cells (ECs) **(A,C)** and WSS by CFD simulation **(B,D)**, scale bar: 2 mm. Green arrows: high ECs concentration, white arrows: high WSS. **(A,B)** 90° angle. **(C,D)** 70° angle.

Figures 2A,C shows ECs distribution on the bottom surface after the 24-h flow exposure experiment with the parallel placement of two struts in the 70 and 90° settings, respectively, with a 3-mm gap. ECs were highly concentrated in the gap and close to the second strut (indicated by green arrows in the figure). As shown in Figures 2B,D, high WSS also appeared in the gap and close to the second strut. This phenomenon was observed in the previous study using one strut (Anzai et al., 2020).

### The Distributions of EC and WSS in the Downstream Area

As shown in Figures 2A,C, after the second strut, ECs were highly concentrated at the downstream area (indicated by green arrows in the figure). This is agreed with the high WSS by CFD simulation in Figures 2B,D.

### Flow Around Struts and EC Distribution on the Strut Surfaces

Focusing on the area around the struts, 70 and 90° displayed a low WSS magnitude symmetrically in both the upstream and downstream areas of both struts as shown in Figures 2B,D. Concerning the WSS distribution around the first and second strut, the 90° setting shows symmetry, whereas the left is higher than the right at 70°.

**FIGURE 3:**
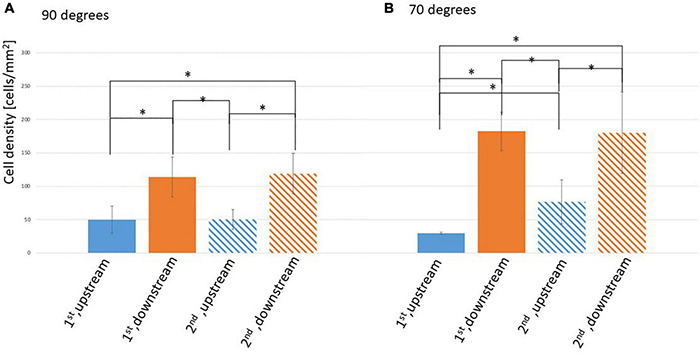
Density of ECs on the surface of the stent strut, two stent struts in a 3-mm gap. **(A)** At 90° angle, mean ± STD, *n* = 6, *p < 0.05 (*t*-test). **(B)** At 70° angle, mean ± STD, *n* = 3, *p < 0.05 (*t*-test).

Figure 3 shows the EC density on the surfaces of the struts in the 90 and 70° settings, respectively. EC density is higher on both downstream surfaces in the 90 and 70° settings compared with those on both upstream surfaces. This density difference on upstream and downstream surfaces of the strut could because of the WSS magnitude difference. The difference between the downstream and upstream surfaces of the 70° setting was larger than that of the 90° setting.

On the side surfaces between the gap (downstream surface of the first strut and upstream surface of the second strut), EC density at the downstream of the first strut was similar to that of the second strut in the 90 and 70° settings. This might be assumed by the WSS similarities with recirculation after the first and the second struts, respectively. In the 70° setting comparing with 90° setting, EC density at the upstream surface of the second strut is higher than its at the upstream surface of the first strut. This may because of the complex flow pattern generated by the tilted angle.

**FIGURE 4:**
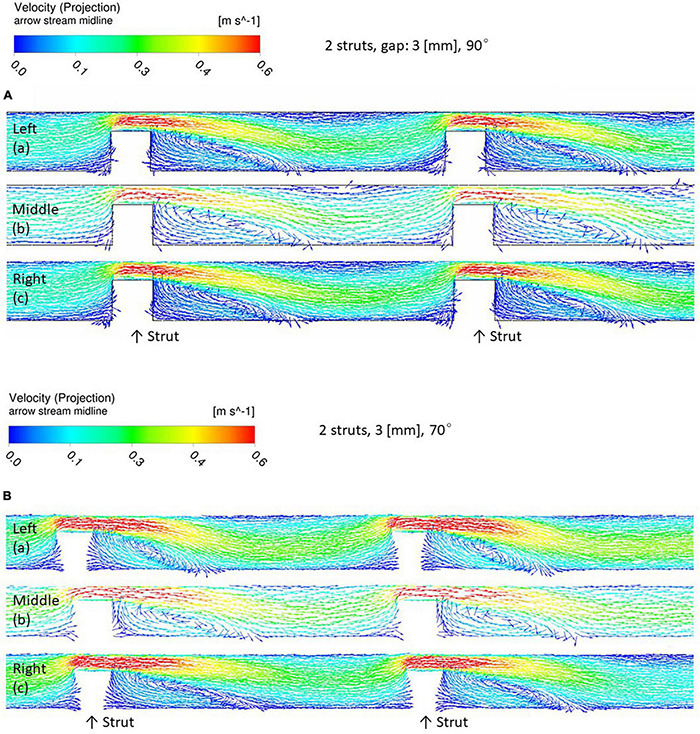
Flow pattern around the two stent struts in 3-mm gap, left, middle, and right parts. **(A)** 90° angle. **(B)** 70° angle.

**FIGURE 5:**
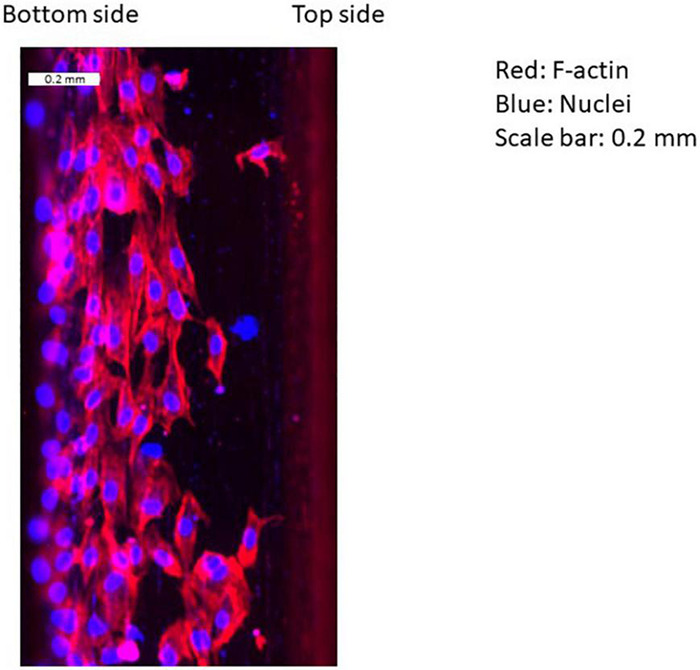
Example of distribution of ECs on the surface of stent strut. Red: F-actin, blue: nuclei, scale bar: 0.2 mm (downstream of the first strut at 90°).

Figure 4 shows flow patterns, indicating two recirculating flows in the gap and after the second strut. Figure 5 shows an example of EC distribution on the strut side surfaces. ECs were highly attached near the bottom side rather than the top. This is because of the cell movement respond to the flow stimuli. At first, there is no cell on the side surface of the strut. After 24 h, ECs migrate on the side surface. Thus, the cell is highly attached from the bottom side.

**FIGURE 6:**
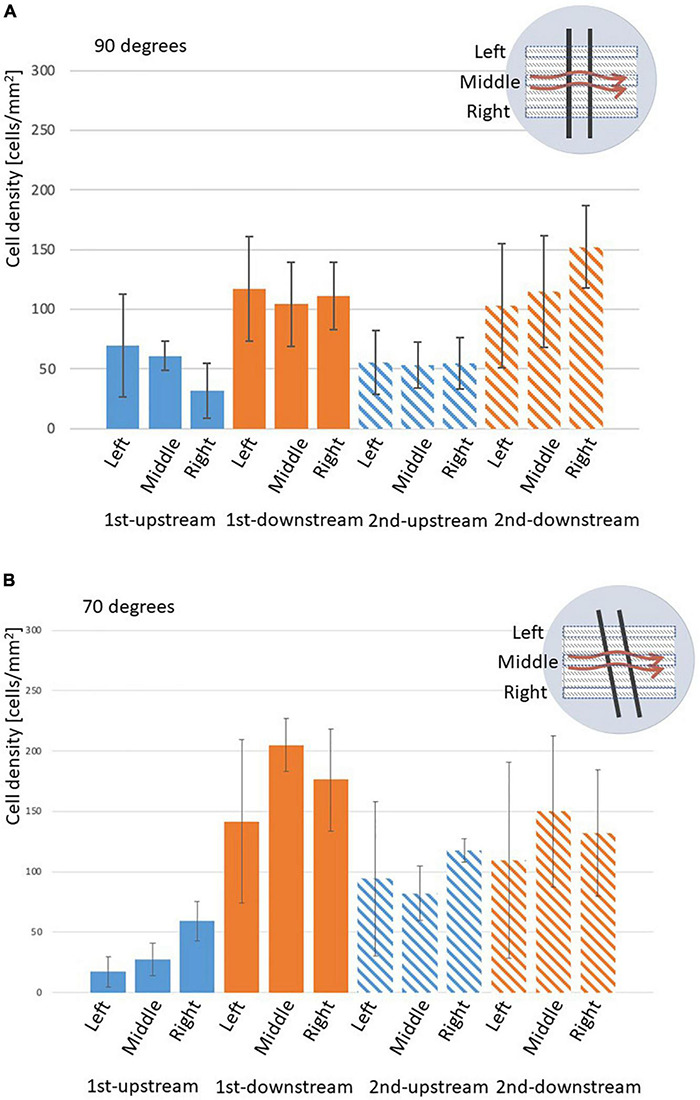
Density of ECs on the surface of stent strut, two stent struts in a 3-mm gap, analyzed on the left, middle, and right parts of the strut, mean ± STD. **(A)** 90° angle, *n* = 6. **(B)** 70° angle, *n* = 3.

In the 90° setting (Figure 4A), the length of the circulating flows was similar in the left, middle, and right. Figure 6 shows EC density variations of the left, middle, and right in struts in the 70 and 90° settings. Figure 6A indicates that, at 90°, ECs on the side surfaces of the gap (the downstream of the first and the upstream of the second struts) were evenly distributed.

In the 70° setting the length extended from the left to the right as shown in Figure 4B. This could be potentially observed as the distance at the top between the strut to the inlet was closer. Therefore, the tilted angle of the struts placement generates complex flow in the gap in the 70° setting. As shown in Figure 6B, in the 70° setting, bigger variations could be observed among the left, middle, and right. This might be explained by the flow patterns highlighted in Figure 4. The recirculation lengths in the left, middle, and right are the same in the 90° setting (Figures 4A, a–c) whereas those of the 70° setting depends on the position of the struts (Figures 4B, a–c). The circulation length in the gap extended in the right compared to that in the left.

### Wall Shear Stress Condition Around the Inlet Area

**FIGURE 7:**
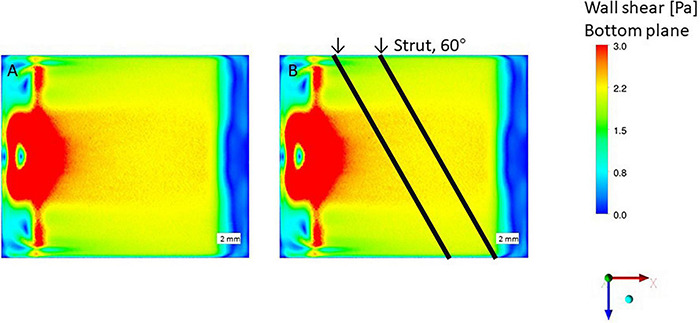
Wall shear stress (WSS) by CFD simulation, no stent strut. **(A)** Wall shear stress condition, without strut. **(B)** Impinging flow affect struts placed in 60-degree.

The WSS condition on the bottom of the flow chamber without struts was 2 Pa as shown in Figure 7A. The results show a high WSS around the inlet area where the impingement of the inflow appears. Figures 2B,D shows the WSS distribution on the bottom surface of the flow chamber with the parallel placement of two struts at 70 and 90° relative to the flow direction with a 3 mm gap. High WSS mainly appears on the center position of the inlet area, caused by the concentrated inflow from the inlet pipe identical to the no strut case (Figure 7). The struts with 70 and 90° did not attach to the high WSS region near the inlet pipe. If two struts with 3 mm gap parallel placed as 60°, complex flow patterns could be generated because the close contact between the strut to the inflow and outflow area.

### Wall Shear Stress Gradient Condition at 90 and 70°

**FIGURE 8:**
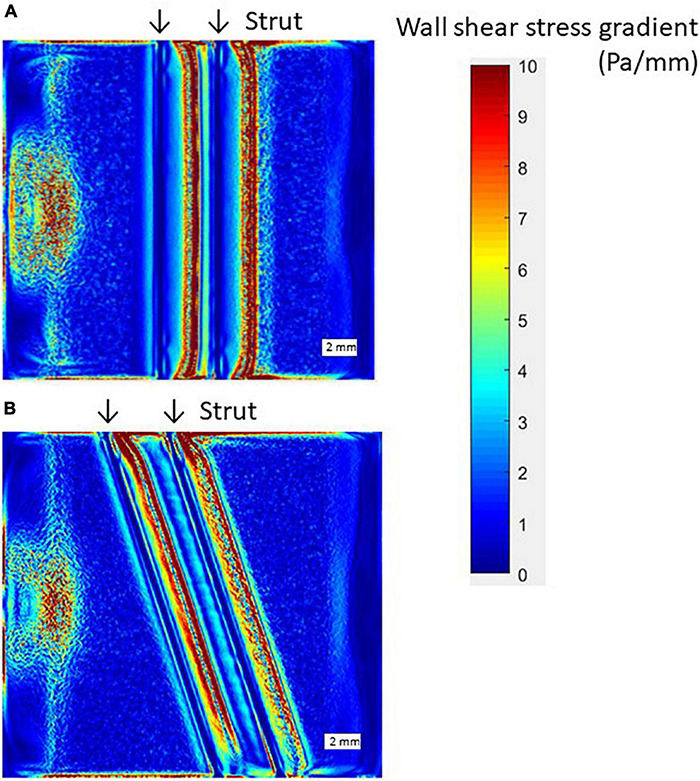
Wall shear stress gradient (WSSG) distribution by CFD simulation, scale bar = 2 mm. **(A)** 90° angle. **(B)** 70° angle.

To investigate the influence of wall shear stress gradient (WSSG) on EC response to flow, WSSG distribution in the flow chamber was analyzed. Figure 8 shows the WSSG on the bottom plane at 90 and 70° (Figures 8A,B, respectively). The region of higher WSSG was almost similar to that of the higher WSS. Therefore, the WSSG effects are not separated from those of the WSS in this chamber with two struts.

## Discussion

This study describes EC distribution after flow exposure with two struts placement. Two struts were set in the flow chamber aiming to reproduce a more realistic situation of stent deployment and can generate complex flow patterns in the gap.

The results revealed that higher cell density at the bottom of the gap could be observed. This might follow the WSS distribution. Moreover, higher EC densities on the downstream surface of the struts were found in both the first and second struts rather than the upstream surfaces. This observation might be related to the influence of the gap between two struts. Furthermore, the cell distribution in the struts varied in the case of the 70° setting. This study reported first on such an observation. These variations are also related to the WSS distribution.

Earlier, Anzai et al. (2020) observed EC distributions after the flow exposure with one stent strut placement. The results in the study revealed that the cell distribution on the bottom had a higher concentration region downstream of the strut. Their study concluded that the cell distribution on the bottom plate could be related to the WSS distribution. In this study, with the presence of gap between two struts, more complex flow patterns were generated in the gap. The cell distribution on the bottom plate also displayed higher concentration regions both in the gap and downstream area. This WSS distribution downstream of the second strut was similar to the one strut results described in the study by Anzai. Furthermore, the cell distribution might also correspond to the WSS distribution in this study.

The region in the gap seemed to be related to the higher WSS. However, the cell distributions between the 90° and 70° settings were not significantly different even though the WSS distributions between the two settings slightly differed. The cell distribution in the gap might be slightly affected by the WSS distribution.

The EC density difference among the left, middle, and right on the side surfaces of strut was observed in this study. On the side surfaced between the gap, the EC density variation difference among the left, middle, and right was higher in the 70° than that in the 90° setting. This was also dependent on the WSS distribution.

In this study, with the presence of two struts, cell migration happened as response to the change of WSS. Due to the WSS change, the WSSG was generated. Yoshino et al. (2017) found a certain influence on the cells, such as morphological response, by the combination of WSS and WSSG with more than 10 Pa and 5 Pa/mm. In this study, the average WSS was 2 Pa on the bottom, and the highest WSSG of 10 Pa/mm appeared. Therefore, the influence of both WSS and WSSG on the EC distribution could exist indeed.

Flow chambers were used for the EC distribution under several flow environments (Dewey et al., 1981; Levesque and Nerem, 1985; Usami et al., 1993; White Charles et al., 2001; Chung et al., 2003; McCann et al., 2005; Sakamoto et al., 2006). The EC responses, such as migration, elongation, and orientation, could be observed in the chamber with WSS generation by the flow environments (Levesque and Nerem, 1985; Sakamoto et al., 2006). Therefore, the use of a chamber with struts under flow environments is a good approach for monitoring EC response. The CFD could indicate flow characteristics such as WSS and the relationship between the flow and EC response could be investigated.

In this study, the difference in EC distribution on the struts could be observed between the proximal and distal walls using a parallel chamber with strut placement. Endothelialization compared to the difference in the distribution on the walls might be affected by the different flows and WSS. These results might contribute to the development of the stent surface for checking endothelialization compared with the no-flow case. Finally, the chamber and CFD combination are also useful to monitor EC response to struts.

### Limitations

This study revealed the EC distribution after flow using a chamber. Chambers are useful to observe cell behaviors. However, the results have several limitations related to the chamber characteristics.

In this study, we analyzed the strut angles of 90° and 70°. The EC distributions on both the bottom plate and strut surface after struts and in the gap between two struts were observed using the degrees combined with WSS, respectively. However, clinical stents on the market exhibit different structures (Lau et al., 2004; Li et al., 2018). We consider that further studies on other angles and gap variations could be performed using a chamber and WSS.

We used only EC monolayers in this study, although the vascular wall exhibits multiple layers. Several researchers have already used a co-culture system with ECs and SMCs (Ziegler et al., 1995; Chiu et al., 2003; Williams and Wick, 2005; Li et al., 2013; Han et al., 2017, 2019) to investigate the signaling pathway between ECs and SMCs under flow conditions. Moreover, the main components of the neo-intimal were overproliferated SMCs controlled by ECs. Han et al. (2017, 2019) found that the change of WSS could affect the expression of SMC protein. However, several studies also used EC layers only and revealed new important findings (Dewey et al., 1981; Levesque and Nerem, 1985; Palmer and Bizios, 1997; Mannino et al., 2015). The use of EC-SMC co-cultures for the evaluation of ECs on the surface could be the subject of future studies.

In this study, the *in vitro* EC response to flow in the chamber could be categorized as the early-stage investigation related to the restenosis process (Miao et al., 2005; Khan et al., 2012). To observe the vascular neo-intimal formation as the late stage, an *in vivo* model would be required that has been used for the evaluation of the late stage (Yue et al., 2008; Kim et al., 2009). In future, the combination of *in vivo* models and *in vitro* chamber with CFD could be potentially useful for the evaluation of EC response on and around the stent.

## Conclusion

This study investigated EC distribution in the presence of two struts, as well as the endothelialization effect on the strut surface. ECs were exposed with two struts placed with a 3-mm gap and 90° or 70° in a chamber. ECs density at the first downstream is higher than that at second upstream. EC density on the downstream surfaces was higher than that on the upstream potentially due to the flow recirculation. Higher ECs density on the bottom at the gap was found following the WSS distributions. Flow disturbance related to the presence of the gap and the strut orientation angle might affect the endothelialization process on the side surfaces of the strut between the gap areas.

## Data Availability Statement

The original contributions presented in the study are included in the article/supplementary material, further inquiries can be directed to the corresponding author/s.

## Author Contributions

ZW performed the cell experiment and wrote manuscript. NP performed CFD simulations. All authors contributed to the article and approved the submitted version.

## Conflict of Interest

The authors declare that the research was conducted in the absence of any commercial or financial relationships that could be construed as a potential conflict of interest. The reviewer AQ declared a past co-authorship with several of the authors HA and MO to the handling editor.

## Publisher’s Note

All claims expressed in this article are solely those of the authors and do not necessarily represent those of their affiliated organizations, or those of the publisher, the editors and the reviewers. Any product that may be evaluated in this article, or claim that may be made by its manufacturer, is not guaranteed or endorsed by the publisher.
